# Fibrodysplasia ossificans progressiva in children: diagnostic pitfalls and *ACVR1* genotype–phenotype spectrum

**DOI:** 10.1007/s00431-026-06974-8

**Published:** 2026-05-02

**Authors:** Nazli Busra Acikgoz, Burcu Senkalfa, Berna Celik Ertas, Zulal Ozdemir Uslu, Ekim Helhel, Gizem Urel Demir, Yasemin Alanay, Adalet Elcin Yildiz, Gulen Eda Utine, Z. Alev Ozon, Pelin Ozlem Simsek Kiper

**Affiliations:** 1 Department of Pediatrics, Division of Pediatric Genetics, Hacettepe University Faculty of Medicine, Ankara, Turkey; 2 Department of Pediatrics, Division of Pediatric Endocrinology and Metabolism, Hacettepe University Faculty of Medicine, Ankara, Turkey; 3 Department of Pediatrics, Division of Pediatric Genetics, Acibadem Mehmet Ali Aydınlar University Faculty of Medicine, Istanbul, Turkey; 4 Department of Radiology, Hacettepe University Faculty of Medicine, Ankara, Turkey

**Keywords:** Fibrodysplasia ossificans progressiva, *ACVR1*, Heterotopic ossification, Genotype–phenotype correlation, Rare disease, Ewing sarcoma

## Abstract

Fibrodysplasia ossificans progressiva (FOP) is a rare multisystem disorder with the triad of congenital hallux valgus, flare-ups and progressive heterotopic ossification. The flare-ups (episodes of painful soft-tissue swelling) start in early childhood, and progress to ossification with cumulative functional disability. We describe the cohort of FOP from a single tertiary center in Türkiye and highlight the phenotypic variabilities and diagnostic pitfalls. The subjects included 10 affected individuals (7 females and 3 males, aged 10 days to 20 years), whose diagnosis was confirmed with molecular tests, including full *ACVR1* sequencing, targeted hotspot analysis, a gene panel for skeletal disorders, and whole-exome sequencing. Clinical and imaging data were retrospectively reviewed. The common pathogenic variant of FOP, c.617G > A, p.(Arg206His) of *ACVR1*, was found in 9 patients, while a rare variant, c.983G > A, p.(Gly328Glu) in one. All patients with the common variant exhibited the classical triad of FOP. Other morbidities included limited cervical mobility in 8, temporomandibular joint involvement in 4, scoliosis in 6, scalp nodules in 6, and brain anomalies in 2. Scalp nodules could be a transient finding with spontaneous regression and were an early manifestation in 2. One patient showed a distinctive brain malformation of FOP (pontine dysmorphism with fusion of the facial colliculi and dorsal bulging into the fourth ventricle). Four patients underwent biopsy prior to referral to our institution. One was coincidentally associated with Ewing sarcoma. The infant with the rare variant presented with limb reduction defects and brain malformation and had not yet developed flare-ups or heterotopic ossification.

*Conclusion*: This single-center cohort from Türkiye confirms the key clinical features of FOP associated with both common and rare *ACVR1* variants and expands the radiological findings with notable features such as pontine malformation. It also highlights scalp nodules as a potential early diagnostic clue, emphasizing the importance of early clinical recognition and timely molecular confirmation, while providing insights that may inform future therapeutic approaches.

**Table:** 

**What Is Known:** *• Fibrodysplasia ossificans progressiva (FOP) is caused by activating variants in ACVR1, most commonly c.617GA, p.(Arg206His), and is characterized by congenital hallux valgus, flare-ups, and progressive heterotopic ossification* *• Early diagnosis is challenging, and misdiagnosis may lead to harmful invasive procedures such as biopsies.* **What Is New:** *• This single-center cohort confirms the key features of FOP, expands the radiological spectrum (including pontine malformation), and identifies scalp nodules as an early diagnostic clue.*

**Supplementary Information:**

The online version contains supplementary material available at 10.1007/s00431-026-06974-8.

## Introduction

Fibrodysplasia ossificans progressiva (FOP, OMIM #135,100) is a rare genetic disorder characterized by congenital bilateral hallux valgus deformity, inflammatory soft-tissue swelling, and progressive heterotopic ossification (HO) [1]. FOP is caused by monoallelic activating variants in *ACVR1*, encoding a BMP type I receptor essential for skeletal and cartilage development. The c.617G > A, p.(Arg206His) variant accounts for more than 97% of cases worldwide [2–5]. The prevalence of FOP is estimated to be 0.04 to 1.36 per million, with approximately 3500–4000 patients worldwide [6, 7].

Affected children start with “flare-ups,” i.e., recurrent episodes of painful axial and paraaxial soft-tissue swelling that typically begin in early childhood. The flare-ups progress to HO, involving the jaw, spine, thoracic cage, and proximal limbs and leading to reduced joint mobility and compromising even respiratory function [2, 8]. Early flare-ups may manifest as transient scalp nodules, especially in infancy [3].

There are no formal clinical diagnostic criteria for FOP. However, when children present with axial or paraaxial soft-tissue swelling or masses, the presence of congenital hallux valgus should raise a high index of suspicion for FOP [2, 3]. The hallux valgus is usually accompanied by a malformed first metatarsal and monophalangism, and is seen in more than 97% of affected individuals. Additional musculoskeletal findings include cervical spine fusion, scoliosis, and increased susceptibility to fractures, which further contribute to long-term disability. Extra-musculoskeletal manifestations, including hearing loss, nephrolithiasis, and limb lymphedema, have also been reported in FOP [3, 4]. Although a few reports have described the coexistence of FOP with malignant tumors, FOP is not considered a cancer-predisposition syndrome [9–11]. Until recently, management of FOP has been limited to supportive care; however, palovarotene, a retinoic acid receptor (RAR)-γ agonist, has been approved by the FDA and is considered a promising disease-modifying therapy, although it may cause premature growth-plate closure in younger patients [12–14].

Data on pediatric FOP in Türkiye are limited. To date, only a few case reports describing children with the common *ACVR1* pathogenic variant have been published. To better characterize the current clinical landscape and healthcare experience of affected individuals in our country, we aimed to provide a comprehensive description of the clinical, molecular, and radiological features of the cohort of 10 patients from a large tertiary center, along with a review of previously reported cases from Türkiye.

## Subjects and methods

This study included 10 patients with FOP, each from an unrelated family, who were evaluated at the Pediatric Genetics outpatient clinic of Hacettepe University Faculty of Medicine (Ankara, Turkey) between 2005 and 2024. Anthropometric measurements were recorded, detailed physical examinations were performed, and all patients underwent radiological evaluation. Standard X-ray imaging was obtained for each patient, and when available, computed tomography (CT), magnetic resonance imaging (MRI), superficial and abdominal ultrasonography, and whole-body SPECT/CT imaging studies were also reviewed. Written informed consent was obtained from the patients or their parents. The study was approved by the Ethics Committee of Hacettepe University Faculty of Medicine (Approval number: SBA 25/671; decision date: 22 July 2025).

Genomic DNA was isolated from peripheral blood using the salt-precipitation method. Full sequencing of the *ACVR1* was performed in 5 patients, targeted hotspot analysis was performed in 3 patients based on clinical suspicion of FOP, a next-generation sequencing (NGS) panel targeting genes associated with skeletal disorders was performed in one patient, and whole-exome sequencing (WES) was performed in one patient whose clinical diagnosis was an unknown malformation syndrome. WES was performed using the Illumina NovaSeq 6000 platform and the Agilent SureSelectXT Human All Exon V8 kit. Raw sequencing data were analyzed using the GRCh38 reference genome.

## Results

### Overview and molecular findings

Ten patients (three males, seven females) were evaluated between 2005 and 2024; ages at presentation ranged from 10 days to 20 years. No other affected individual was reported in any of the families. Their manifestations are summarized in Table 1 and Supplementary Information (SI) 1. All patients harbored pathogenic variants in *ACVR1* according to ACMG criteria.

**Table 1:** Clinical, demographic, and phenotypic characteristics of ten patients with fibrodysplasia ossificans progressiva (FOP). The table summarizes age at symptom onset and evaluation, sex, family history, anthropometric measurements, major skeletal manifestations, soft-tissue findings, functional limitations, associated clinical features, treatment history, and outcomes

Feature	Patient 1	Patient 2	Patient 3	Patient 4	Patient 5	Patient 6	Patient 7	Patient 8	Patient 9	Patient 10
Age at symptom onset/initial symptoms	2 years/Scalp nodules	3 years/Scalp nodules	11 months/soft-tissue swellings	6 years 5 months/soft-tissue swellings	8 years 6 months/soft-tissue swellings	Present at birth/Hallux valgus malformations	11 years/trauma-induced heterotopic ossification	5 years 4 months/heterotopic ossification	Present at birth/prenatally detected agenesis of the corpus callosum	2 years 3 months/soft-tissue swellings
Age at first evaluation in our clinic	3 years 9 months	20 years	6 years 4 months	10 years	8 years 6 months	10 days	12 years	6 years 6 months	4 months	2 years 7 months
Sex	F	M	F	F	M	F	F	M	F	F
Parental consanguinity (2/10)	-	+	-	-	+	-	-	-	-	-
Height (cm, SDS)	104 cm (1.01 SDS)	169 cm (− 1.07 SDS)	119.5 cm (0.37 SDS)	141.5 cm (0.59 SDS)	127.5 cm (− 1.26 SDS)	N/A	165 cm (1.78 SDS)	124.5 cm (1.06 SDS)	58 cm (− 1.4 SDS)	105 cm (3.38 SDS)
Weight (kg, SDS)	18 kg (1.08 SDS)	67 kg (− 0.22 SDS)	20 kg (− 0.4 SDS)	36 kg (0.5 SDS)	27 kg (− 0.58 SDS)	3.14 kg (− 1.06 SDS)	47 kg (0.51 SDS)	26 kg (1.06 SDS)	5.5 kg (− 0.87 SDS)	18 kg (2.54 SDS)
HC (cm, SDS)	47.5 cm (0.84 SDS)	57 cm (− 0.47 SDS)	51 cm (− 0.15 SDS)	54 cm (0.75 SDS)	52.5 cm (− 0.47 SDS)	35 cm (− 0.38 SDS)	52 cm (− 1.51 SDS)	54 cm (1.44 SDS)	41 cm (0.08 SDS)	49 cm (0.38 SDS)
Hallux valgus malformations (9/10)	+	+	+	+	+	+	+	+	-	+
Heterotopic ossification (on physical examination) (9/10)	+	+	+	+	+	+	+	+	-	+
Soft-tissue swellings (on physical examination) (9/10)	+	+	+	+	+	+	+	+	-	+
Scalp nodules (on physical examination) (6/10)	+	+	+	+	-	+	-	+	-	-
Osteochondromas (6/8)	N/A	+	+	N/A	+	+	+	+	-	-
Dental/oral findings (5/10)	-	High-arched and narrow palate, limited TMJ mobility, dental crowding	-	High-arched palate, limited TMJ mobility	High-arched palate	Limited TMJ mobility	-	High-arched and narrow palate, limited TMJ mobility	-	-
Scoliosis (6/10)	+	+	+	-	+	+	+	-	-	-
Chest deformities (3/10)	-	-	+	-	-	+	-	+	-	-
Distal limb reduction defects (1/10)	-	-	-	-	-	-	-	-	+	-
Restricted neck mobility (on physical examination)(8/10)	+	+	+	+	+	+	-	+	-	+
Limitation of joint mobility (on physical examination)(9/10)	+	+	+	+	+	+	+	+	-	+
Hearing loss	-	? (History of eight ventilation tube placements)	-	-	-	-	-	-	-	-
Renal stones (2/10)	-	-	-	+	-	-	-	-	-	+
Cognitive state	N	N	N	N	N	N	N	N	N	N
Dysmorphic facial features (9/10)	Prominent nasal bridge	Wide forehead, downslanting palpebral fissures, retrognathia	Retromicrognathia	Upslanting palpebral fissures, prominent nasal bridge, high-arched palate	Upslanting palpebral fissures, retrognathia	Upslanting palpebral fissures, open bite	Alopecia areata, Upslanting palpebral fissures	Retrognathia, high-arched and narrow palate	Hypertelorism, bilateral epicanthus, anteverted ears, abnormal helix folding of the left ear, triangular philtrum, thin upper lip, sparse eyebrows and eyelashes	No facial dysmorphism
Neuroradiological findings (3/8)	-	N/A	-	-	-	Brainstem hypoplasia, polymicrogyria, and demyelinating lesions in the white matter and dentate nucleus	N/A	Pontine dysmorphism with fusion of the facial colliculi and dorsal surface bulging into the fourth ventricle	Agenesis of corpus callosum, mega cisterna magna, and colpocephaly	-
Biopsy (5/10)	-	A spindle cell lesion with low mitotic index, infiltrating the adjacent adipose tissue	Benign myofibroblastic proliferation	Myositis ossificans?	-	-	Low-grade osteochondral dysplasia	-	-	Due to suspected malignancy
Other findings	Hypertrichosis	-	-	Myopia	-	-	-	-	-	Restrictive lung disease, SIADH, died at 14 years due to Ewing sarcoma
Regular follow-up (7/10)	-	-	+	-	+	+	+	+	+	+
*ACVR1 variant (NM_001111067.4)*	c.617G > A p.Arg206His	c.617G > A p.Arg206His	c.617G > A p.Arg206His	c.617G > A p.Arg206His	c.617G > A p.Arg206His	c.617G > A p.Arg206His	c.617G > A p.Arg206His	c.617G > A p.Arg206His	c.983G > A p.Gly328Glu	c.617G > A p.Arg206His
Segregation analysis (7/10)	De novo	N/A	De novo	De novo	De novo	De novo	N/A	De novo	De novo	N/A

Abbreviations: CT, computed tomography; F, female; FOP, fibrodysplasia ossificans progressiva; HC, head circumference; M, male; MRI, magnetic resonance imaging; N, normal; N/A, not available; SDS, standard deviation score; SIADH, syndrome of inappropriate antidiuretic hormone secretion; TMJ, temporomandibular joint; US, ultrasonography

**Fig. 1:**
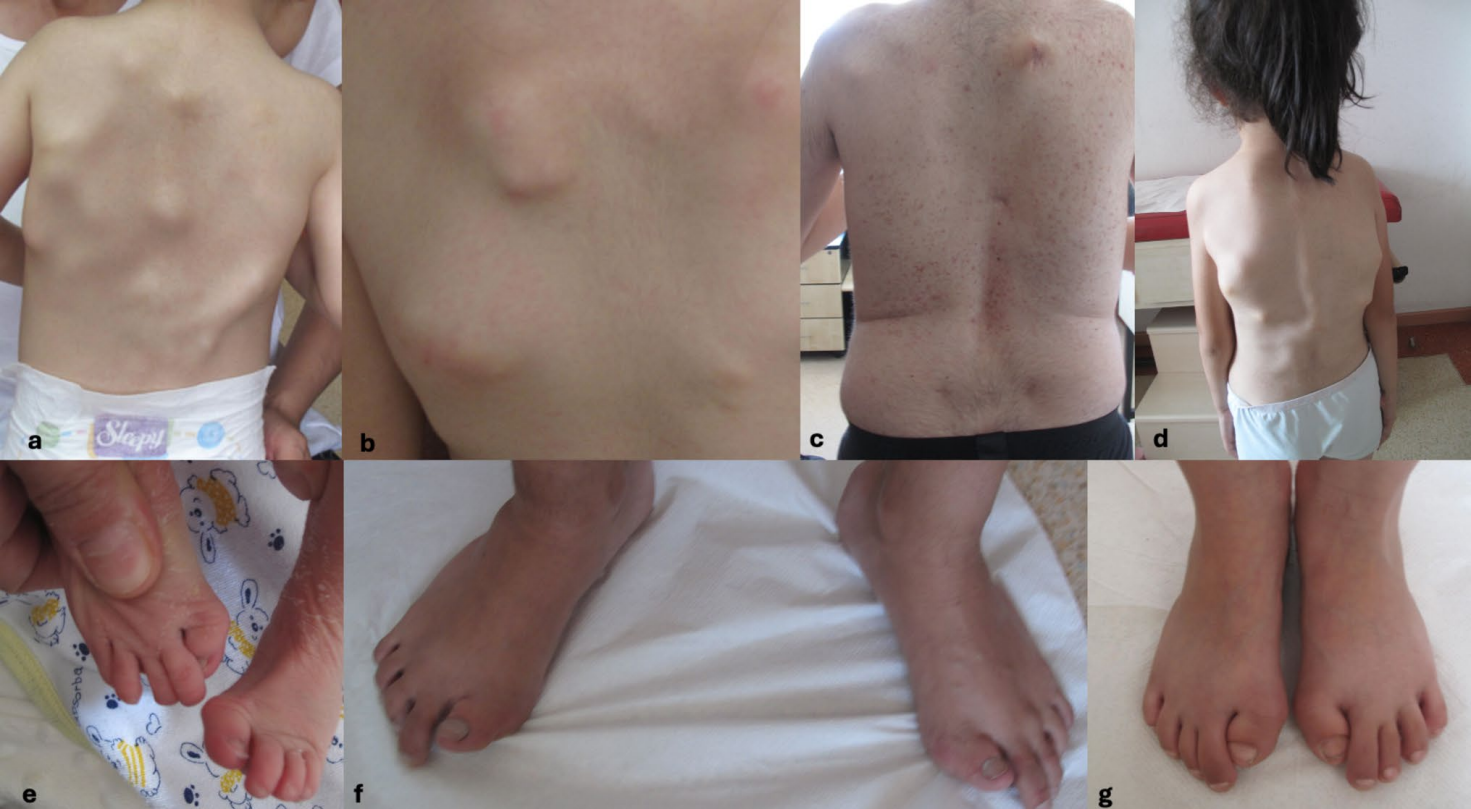
Clinical photographs of patients with fibrodysplasia ossificans progressiva (FOP). **a**–**d** Representative examples of inflammatory soft-tissue swellings (flares) involving the trunk/back (Patients 6, 4, 2, and 3, respectively). **e**–**g** Congenital great toe malformations characteristic of FOP (Patients 6, 2, and 3, respectively)

**Fig. 2:**
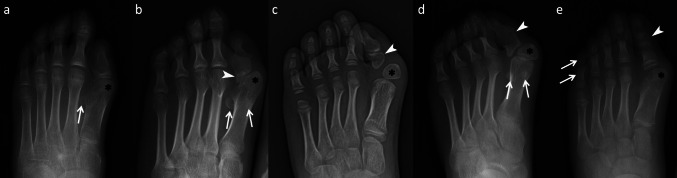
Radiographs of great toe malformations in fibrodysplasia ossificans progressiva (FOP) showing monophalangeal or biphalangeal hallux. **a** A 9-year-old female (Patient 10) with a monophalangeal great toe, mildly medially deviated malformed metatarsal head (asterisk), and hallucal sesamoids; the fibular sesamoid (arrowhead) is visible, whereas the tibial sesamoid is not. **b** A 20-year-old male (Patient 2) with mild hallux valgus and a monophalangeal great toe, showing a medially deviated malformed metatarsal head (asterisk), hallucal sesamoids (arrows), and an ectopic ossification center (EOC) (arrowhead). **c** A 6-year-old female (Patient 7) with a biphalangeal hallux and a proximal “delta phalanx” (arrowhead) with an asymmetric amorphous shape; an EOC (asterisk) is located at the distal-medial aspect of the hallux metatarsal head, with moderate hallux valgus. **d** Follow-up radiography at age 12 years of the same patient shown in **c**, demonstrating complete fusion of the first-digit phalanges (arrowhead), whereas the EOC (asterisk) persists; mild hallucal sesamoids are present. **e** A 9-year-old male (Patient 5) with a biphalangeal hallux and mild hallux valgus. Note the enlarged secondary ossification center (arrowhead) of the distal phalanx, the medially deviated malformed metatarsal head (asterisk), and a biphalangeal fifth digit (arrows)

Nine patients presented with the classical features of FOP, i.e., hallux valgus malformations, soft-tissue swellings, and HO (Figs. 1 and 2). They underwent sequencing analysis for *ACVR1* in accordance with the clinical diagnosis of FOP, which revealed the common heterozygous missense variant of FOP c.617G > A, p.(Arg206His) in exon 6.

One patient (Patient 9), a 4-month-old infant, had terminal reduction of the fingers and toes, agenesis of the corpus callosum, mega cisterna magna, and colpocephaly, which were detected on fetal ultrasound. The infant has not yet developed flare-ups or HO to date. WES identified a heterozygous missense variant c.983G > A, p.(Gly328Glu) in exon 8 (Table 1).

Segregation analysis was performed in 7 families, confirming the variant as de novo. In the remaining three patients, segregation analysis could not be performed.

### Manifestation of 9 patients with the common pathogenic variant

#### Clinical findings

Initial symptoms included scalp nodules in 2, soft-tissue swellings in 4, congenital hallux valgus malformation in one patient, and HO in 2 patients. At presentation and during follow-up, height, weight, and head circumference measurements were within the − 2 to + 2 SDS range in all patients but one (Patient 10) who exhibited height and weight measurements above + 2 SDS, and developed Ewing sarcoma at 12 years of age and died at 14 years.

Late consequences included restricted neck mobility in 8, limitation of temporomandibular joint (TMJ) movement in 4, and limitation of joint mobility involving multiple joints in different anatomical regions and with variable severities in 9 patients. Scoliosis was present in 6 patients. Prior to diagnosis, 4 patients had a history of unnecessary biopsies.

#### Radiologic findings

**Fig. 3:**
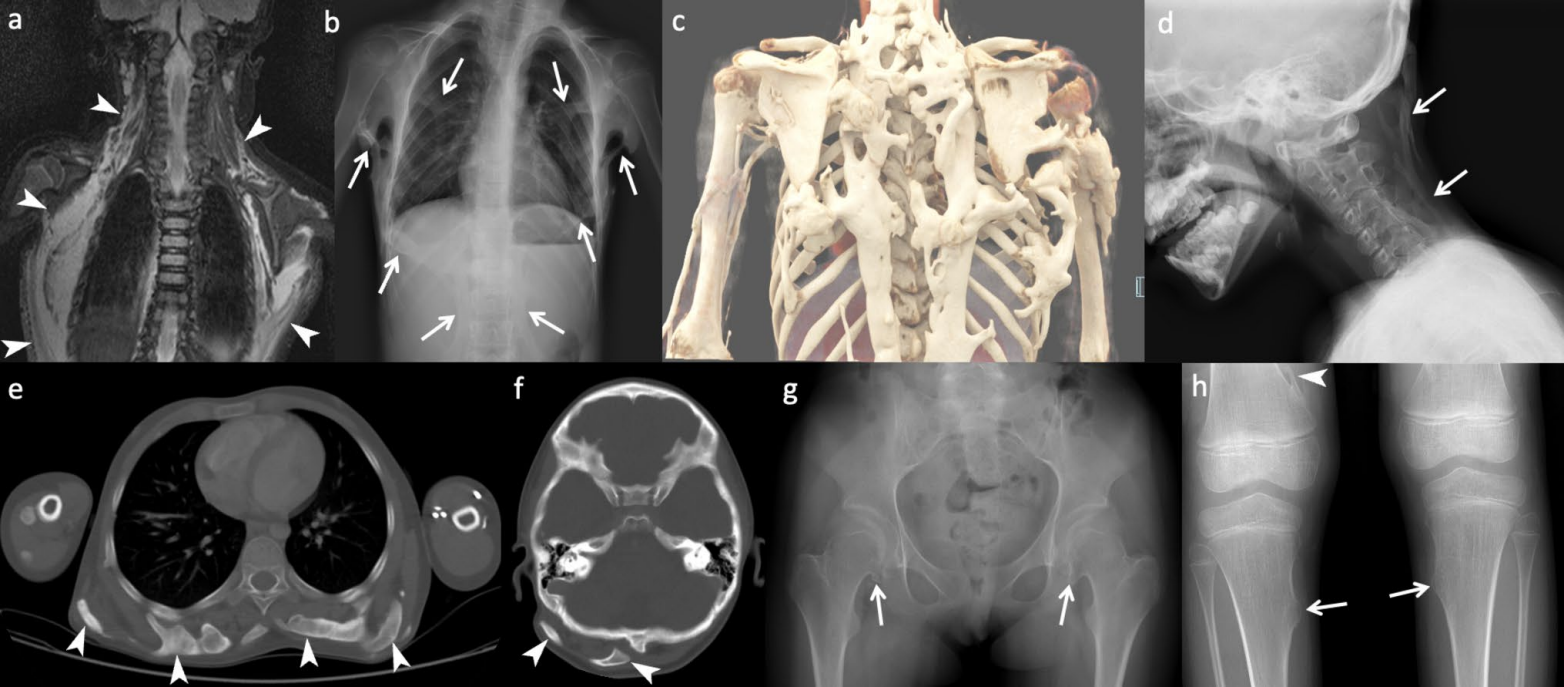
Imaging findings in fibrodysplasia ossificans progressiva (FOP). **a** Coronal short tau inversion recovery (STIR) image of a 13-month-old female (Patient 3) showing extensive myofascial edema (arrowheads) in the posterolateral thoracic wall at an early disease stage. **b** Follow-up radiography at age 6 years in the same patient demonstrates progression to heterotopic ossification of the thoracic wall, paraspinal, and axillary regions (arrows). **c** Three-dimensional volume-rendered image of an 8-year-old female (Patient 4) showing extensive, irregular ossifications involving the thoracic wall, paraspinal regions, and upper extremities. **d** Lateral cervical spine radiography of a 5-year-old male (Patient 8) demonstrating nuchal membrane ossification (arrows) and cervical facet joint ankylosis from C2 to C7, with enlarged posterior elements and hypoplastic vertebral bodies. **e**, **f** Axial CT images of the thorax (**e**) and head (**f**) from the same patient as in (**d**) showing thick subcutaneous, fascial, and/or intramuscular ossifications (arrowheads); the scalp lesion corresponds to a clinically described scalp nodule. **g** Anteroposterior (AP) pelvis radiography of the same patient as in **a** showing short, broad femoral necks with hypoplastic femoral heads, acetabular dysplasia, and small osteochondromas (arrows) at the medial femoral necks. **h** AP knee radiographs of another 6-year-old female (Patient 7) showing small sessile osteochondromas (arrows) of the proximal medial tibiae and a pedunculated osteochondroma (arrowhead) of the right distal femur

Radiological findings are summarized in SI 1. All patients showed the classical skeletal changes, i.e., hallux valgus deformities with phalangeal dysmorphism and variable pseudoepiphyses and HO most frequently involving the aponeuroses, fascia, and skeletal muscles and variably the tendinous regions (Fig. 3 and SI 1). The anatomical distribution of HO is illustrated in SI 2. Flare-ups or inflammatory soft-tissue masses were also seen predominantly in the aponeuroses, fascia, and skeletal muscles, with occasional involvement of subcutaneous and deep soft tissues. Notably, scalp nodules were detected in 6 patients, likely reflecting their transient nature and/or spontaneous resolution. Additional radiological findings included osteochondromas-like bone projection, scoliosis, cervical spine fusions, short, broad femoral necks with hypoplastic femoral heads, acetabular dysplasia, and osteoporotic changes (Fig. 3 and SI 1).

#### Brain anomalies

**Fig. 4:**
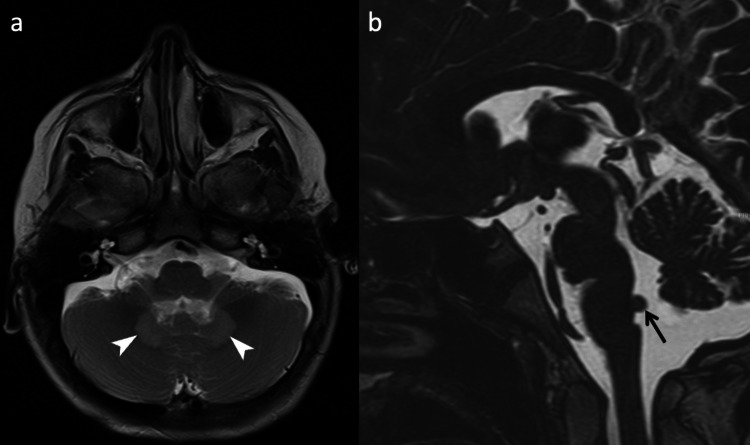
Neuroimaging findings in fibrodysplasia ossificans progressiva (FOP). **a** Axial T2-weighted magnetic resonance imaging (MRI) of a 19-month-old female (Patient 6) showing dentate nucleus hyperintensity (arrowheads). **b** Sagittal T2-weighted MR image of a 9-year-old male (Patient 8) demonstrating pontine dysmorphism with fusion of the facial colliculi and dorsal surface bulging into the fourth ventricle (arrows)

Central nervous system anomalies were found in 2 patients, who showed brainstem hypoplasia, demyelinating lesions, and polymicrogyria. Distinctively, one patient demonstrated pontine dysmorphism with fusion of the facial colliculi and dorsal bulging into the fourth ventricle (Fig. 4 and SI 1).

#### Other extra-musculoskeletal manifestations

Most individuals showed minor facial dysmorphism, including retrognathia and upslanting palpebral fissures. Hearing loss was documented in only one patient (patient 2), who had undergone multiple ventilation tube insertions but could not undergo formal audiological evaluation. Nephrolithiasis was present in 2 patients.

## Discussion

To date, only a few case reports on FOP patients with the common p.Arg206His variant and typical clinical course have been published from Türkiye [15–19], yet comprehensive data remain limited (SI 3). This study represents a relatively large cohort from a single-center in our country. In general, the observations in this cohort recapitulated the classical clinical features of FOP (e.g., bilateral hallux valgus deformities, flare-ups, and HO), and also reaffirmed that progressive limitation of joint mobility (e.g., restricted temporomandibular joint mobility, ankylosis of the cervical spine, scoliosis, and thoracic cage deformities) leads to striking physical morbidity of affected individuals [20, 21].

Nonetheless, our experiences included less well-recognized findings, reflecting the broad phenotypic spectrum of FOP [3, 4, 12, 22]. Scalp nodules were prevalent and may be a temporary finding. Radiographic findings that are less frequently reported included mild acetabular dysplasia, short/thick femoral necks, and osteoporotic changes, which further underscore the widespread skeletal involvement in FOP. Awareness of atypical findings in a rare *ACVR1* variant, c.983G > A, p.(Gly328Glu), is important to avoid potential diagnostic errors.

Moreover, brain malformation in FOP may deserve special comments. Structural brain anomalies that have been reported in FOP include dentate nucleus T2 hyperintensities, brainstem dysgenesis, and corpus callosum hypoplasia [22–25]. Our neuroimaging findings (i.e., brainstem hypoplasia, colpocephaly, demyelinating lesions, polymicrogyria, and corpus callosum agenesis with ventricular anomalies) further expand this spectrum. A notable finding was pontine dysmorphism with fusion of the facial colliculi and dorsal bulging into the fourth ventricle, which was previously reported as a part of a broader spectrum of pontine and other CNS abnormalities in FOP [26]. Some studies reported cognitive delay in FOP patients [3], while individuals in our cohort did not show cognitive dysfunction, albeit a formal cognitive assessment was not carried out. Brain malformation in FOP can exist, even though affected individuals are neurologically asymptomatic. The pathogenesis of central nervous system involvement in FOP remains incompletely understood, and this neurologic issue requires further investigation [23].

Individuals with FOP may show minor facial dysmorphism, including retrognathia, a flattened nasal bridge, and sparse hair [27]. However, these features are not always characteristic or consistent across patients. In our cohort, a wide spectrum of craniofacial findings was observed, highlighting the phenotypic variability of FOP. Likewise, some extra-musculoskeletal abnormalities, including hearing impairment, lymphedema, and nephrolithiasis, may be infrequent comorbidities in FOP [12, 28]. Although these features were not dominant in our cohort, careful monitoring for these comorbidities remains clinically relevant during longitudinal follow-up.

It is noteworthy that 4 patients underwent biopsy for flare-ups prior to referral to our department. The invasive procedure poses a significant risk of disease exacerbation. Given the irreversible nature of HO triggered by trauma or surgical manipulation, biopsy is strictly contraindicated in suspected FOP cases [12]. Awareness of the distinctive malformation of the great toe can prevent the malpractice.

Patient 10 underwent biopsy after a confirmed diagnosis of FOP because of suspected malignancy, which subsequently revealed Ewing sarcoma. FOP is not considered a cancer-predisposition syndrome and malignant transformation is not recognized as a feature of the disease [10]. To our knowledge, only isolated malignant tumors have been described in a few individuals with FOP, such as a case with basal cell carcinoma treated with radiotherapy [10]. The diagnosis of Ewing sarcoma in Patient 10 was interpreted as a likely coincidental finding. Nevertheless, this case highlights an important diagnostic challenge to distinguish a coincidental malignant tumor from an inflammatory swelling during repeated flare-ups. The important task largely rests on diagnostic imaging, including MRI, CT, ultrasonography, and whole-body SPECT/CT imaging. Although these imaging modalities can identify inflammatory and ossification-related changes of flare-ups, their findings are often non-specific and must be interpreted, alongside clinical features, for a reliable diagnosis [21, 29].

**Fig. 5:**
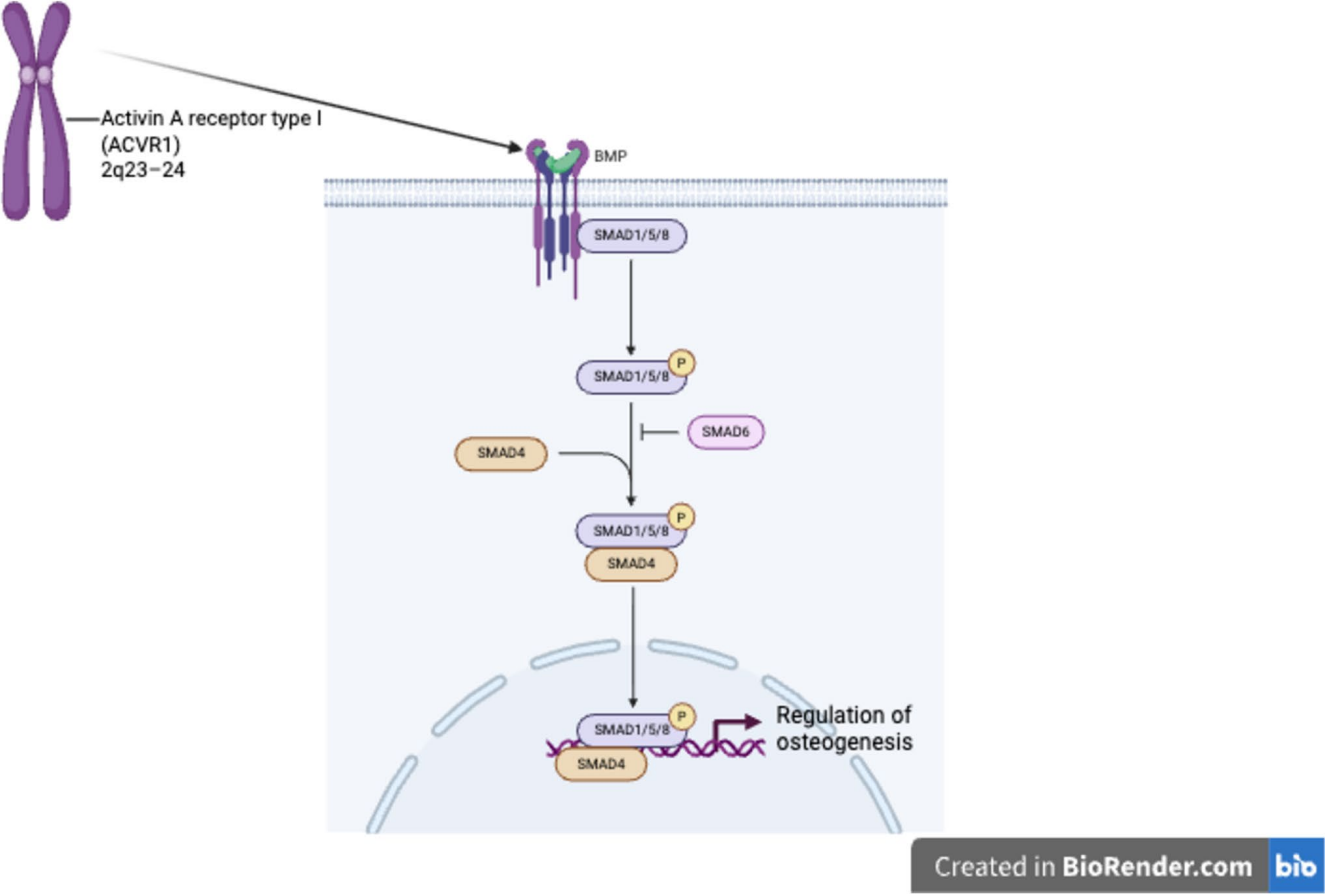
Schematic representation of *ACVR1*-mediated BMP signaling. Upon binding of BMP ligands, *ACVR1* activates downstream SMAD1/5/8 signaling, which forms a complex with SMAD4 and translocates to the nucleus to regulate osteogenic gene expression. Created with BioRender.com

The pathogenic link between *ACVR1* and FOP was identified in 2006 [30]. The gene is located on chromosome 2q23–24, and encodes a type I BMP receptor belonging to the TGF-β receptor superfamily. This receptor plays a crucial role in a wide range of biological processes, including the development and regulation of the skeletal, cardiac, cartilaginous, neural, and reproductive systems [31]. ACVR1 binds to activin A as well as several BMP ligands and mediates downstream signaling primarily through the SMAD1/5/8 pathway [32]. In addition, it can activate non-canonical pathways such as MAPK and PI3K/AKT [31]. A schematic representation of *ACVR1*-mediated signaling pathways is shown in Fig. 5.

To date, 28 pathogenic or likely pathogenic *ACVR1* variants linked to FOP have been described in ClinVar, including L196P, P197_F198delinsL, R202I, Q207E, F246, R258S, R258G, G325A, G328E, G328R, G328W, and G356D. These variants are associated with variable clinical features, differences in disease severity and age of onset [31].

Over 95% of affected individuals have c.617G > A, p.(Arg206His) (R206H), that is located within the intracellular glycine-serine-rich (GS) domain of the receptor [33]. Individuals with the common pathogenic variant present with classical FOP phenotypes. As with Patient 9 in this cohort, the p.Gly328Glu (G328E) variant has been reported to show an earlier and more severe disease course as well as distal limb reduction defects affecting all digits with syndactyly and anonychia, and brain malformation (agenesis of the corpus callosum, mega cisterna magna, and colpocephaly) [3, 21, 34–38]. This rare variant occurs outside the GS region in the kinase catalytic domain adjacent to the GS-rich loop, and may alter local conformation and electrostatic interactions, potentially disrupting the inactive state of the kinase [34]. Patients with the rare variant may show cognitive impairment, craniofacial dysmorphisms, and sparse eyebrows, or alopecia partialis [3].

## Conclusion

This study represents a single-center cohort of genetically confirmed FOP patients from Türkiye. Our findings reaffirm the clinical hallmarks of FOP and broaden the phenotypic spectrum, including central nervous system anomalies in association with both common and rare A*CVR1* variants. Given the irreversible nature of HO, early clinical recognition and timely molecular confirmation are essential for optimal management and genetic counseling, and may also contribute to the development of future therapeutic approaches.

## Limitations

This study has some inherent limitations. The relatively small number of patients restricts the extent to which the findings can be generalized to the broader FOP population. In three cases, segregation analysis could not be performed due to the unavailability of parental samples. In addition, follow-up duration varied between patients and may not have been sufficient to comprehensively capture long-term disease progression. Further longitudinal studies with larger cohorts, extended follow-up, and standardized outcome measures are needed to better characterize the natural history and variability of FOP.

## Supplementary Information

Below is the link to the electronic supplementary material.

ESM 1 (DOCX 49.8 KB)

ESM 2 (DOCX 1.59 MB)

ESM 3 (DOCX 23.9 KB)

## Data Availability

No datasets were generated or analysed during the current study.

## Abbreviations

ACVR1: Activin A receptor type I
BMP: Bone morphogenetic protein
CNS: Central nervous system
CT: Computed tomography
FOP: Fibrodysplasia ossificans progressiva
HO: Heterotopic ossification
MRI: Magnetic resonance imaging
NGS: Next-generation sequencing
SDS: Standard deviation score
SPECT/CT: Single-photon emission computed tomography/computed tomography
STIR: Short tau inversion recovery
TMJ: Temporomandibular joint
WES: Whole-exome sequencing
