# Supplementary material for: Fibrodysplasia ossificans progressiva in children: diagnostic pitfalls and ACVR1 genotype–phenotype spectrum
Source: Eur J Pediatr. 2026 May 2;185(5):335. doi: 10.1007/s00431-026-06974-8 (PMC13135564; doi:10.1007/s00431-026-06974-8)
Supplement: Supplementary file 1 — (DOCX 49.8 KB) [file 431_2026_6974_MOESM1_ESM.docx]

| Supplementary Information (SI) 1. Radiological findings in ten patients with fibrodysplasia ossificans progressiva (FOP). | | | | | | | |  |  |  |  |  |
| --- | --- | --- | --- | --- | --- | --- | --- | --- | --- | --- | --- | --- |
|  | **Feature** |  | **Patient 1** | **Patient 2** | **Patient 3** | **Patient 4** | **Patient 5** | **Patient 6** | **Patient 7** | **Patient 8** | **Patient 9** | **Patient 10** |
| **Skeletal Anomalies** |  | Clinodactyly | N/A | - | - | N/A | N/A | - | - | + | N/A | + |
|  |  | Pseudoepiphysis | - | - | - | - | - | + | + | + | N/A | - |
|  |  | Hallux valgus | + | + | + | + | + | + | + | + | N/A | + |
|  |  | Phalangeal dysmorphism (+/-) | + | + | + | N/A | + | + | + | + | N/A | + |
|  |  | Monophalangism | N/A | + | + | N/A | - | + | + | + | N/A | + |
|  |  | Biphalangism | N/A | - | - | N/A | + | - | - | - | N/A | - |
|  |  | Phalangeal fusion (symphalangism) | N/A | - | - | N/A | - | - | - | - | N/A | - |
|  |  | Metatarsal dysmorphism (+/-) | N/A | + | + | N/A | + | + | - | - | N/A | + |
|  |  | Secondary ossification center dysmorphism (+/-) | N/A | - | + | N/A | + | + | + | + | N/A | - |
|  |  | Ectopic ossification center | N/A | + | - | N/A | - | - | - | - | N/A | - |
|  |  | Hallucal sesamoid bones | N/A | + | - | N/A | - | - | + | - | N/A | - |
|  |  | Lateral epiphyseal bracket | N/A | - | - | N/A | - | - | - | - | N/A | - |
|  |  | Digit 2-5 dysmorphism | N/A | 3rd metatarsal head dysmorphic | - | N/A | 5th digit biphalangism | - | - | 2-4 proximal phalanges dysmorphic | N/A | - |
|  | Involved region | Head and neck | + | + | + | N/A | + | + | N/A | + | - | - |
|  |  | Axillary shoulder | + | + | + | + | + | + | + | + | - | + |
|  |  | Thoracic wall | - | + | + | + | + | + | + | + | - | + |
|  |  | Abdominal wall | - | - | - | - | - | + | + | + | - | - |
|  |  | Paraspinal | + (whole) | + | + | + | + | + | + | + | - | + |
|  |  | Pelvis | N/A | + | - | + | - | + | + | - | - | + |
|  |  | Upper extremity | N/A | + | N/A | + | - | + | + | + | - | + |
|  |  | Lower extremity | N/A | + | + | N/A | + | - | + | - | - | - |
|  | Heterotopic ossification  **Skeletal Anomalies** | Aponeuroses | - | + | + | + | + | + | + | + | - | - |
|  |  | Fascia | - | + | + | + | + | + | + | + | - | + |
|  |  | Ligaments | - | - | + | + | - | + | - | - | - | - |
|  |  | Tendons | - | + | + | + | - | + | + | - | - | + |
|  |  | Skeletal muscles | - | + | + | + | + | + | + | + | - | + |
|  | Inflammatory soft-tissue swellings | Aponeuroses | + | N/A | + | + | + | + | + | N/A | N/A | N/A |
|  |  | Fascia | + | N/A | + | + | + | + | + | N/A | N/A | N/A |
|  |  | Ligaments | - | N/A | + | + | - | - | - | N/A | N/A | N/A |
|  |  | Tendons | + | N/A | + | + | - | - | - | N/A | N/A | N/A |
|  |  | Muscles | + | N/A | + | + | + | + | + | N/A | N/A | N/A |
|  |  | Subcutaneous and deep soft tissue | + | N/A | + | + | - | + | + | N/A | N/A | N/A |
|  | Scalp nodules |  | + | N/A | - | + | - | + | N/A | + | - | - |
|  | Osteochondromas | Proximal medial tibia | N/A | - | + | N/A | + | + | + | + | - | - |
|  |  | Other localization | - | + femur | + femur | N/A | + clavicle and humerus | - | + femur | + femur | - | - |
|  | Osteoarthritis |  | - | - | - | - | - | - | - | - | - | - |
|  | Cervical spine fusions | Large posterior elements | - | - | + | + | + | + | - | + | + | + |
|  |  | Tall narrow vertebral bodies | - | - | + | + | + | + | - | + | + | + |
|  |  | Variable fusion of the facet joints between C2 and C7 | - | + | + | - | - | + | - | + | - | + |
|  | Thoracolumbar fusions |  | - | + | - | - | - | + | - | - | - | + |
|  | Malformation of the temporomandibular joints |  | - | N/A | N/A | - | - | N/A | N/A | - | - | - |
|  | Fusions of the costovertebral and costotransverse joints  **Skeletal Anomalies** |  | - | - | N/A | - | - | - | - | - | - | - |
|  | Chest deformities |  | - | - | + | - | - | + | - | + | - | - |
|  | Short, broad femoral neck |  | N/A | + | + | + | + | + | + | + | + | + |
|  | Acetabular dysplasia |  | N/A | + | - | + | - | - | + | + | - | + |
|  | Scoliosis |  | + | + | + | - | + | + | + | - | - | - |
|  | Distal limb reduction defects |  | N/A | - | - | N/A | - | - | - | - | + | - |
|  | Osteopenia |  | + | + | + | + | + | + | + | + | - | + |
|  | Fractures |  | - | - | - | + | - | - | - | - | - | + (pathologic) |
|  | Other |  |  |  |  |  |  |  |  | First metacarpal shortening |  | Right femur Ewing sarcoma |
|  | Agenesis of corpus callosum |  | N/A | N/A | - | - | - | - | N/A | - | + | - |
|  | Demyelinating lesions  **Central Nervous System Anomalies** | White matter | N/A | N/A | - | N/A | - | + | N/A | - | N/A | - |
|  |  | Dentate nucleus | N/A | N/A | - | N/A | - | + | N/A | - | N/A | - |
|  |  | Spinal cord | - | N/A | - | N/A | - | N/A | N/A | N/A | N/A | - |
|  | Cortical atrophy |  | N/A | N/A | - | - | - | - | N/A | - | N/A | - |
|  | Hippocampal atrophy |  | N/A | N/A | - | N/A | - | - | N/A | - | N/A | - |
|  | Cerebellar anomalies |  | N/A | N/A | - | N/A | - | - | N/A | - | N/A | - |
|  | Mega cisterna magna |  | N/A | N/A | - | - | - | - | N/A | - | + | - |
|  | Colpocephaly |  | N/A | N/A | - | - | - | - | N/A | - | + | - |
|  | Polymicrogyria |  | N/A | N/A | - | N/A | - | + | N/A | - | N/A | - |
|  | Brainstem hypoplasia |  | N/A | N/A | - | - | - | + | N/A | + | N/A | - |
|  | Hydrocephalus |  | N/A | N/A | - | - | - | - | N/A | - | N/A | - |

Abbreviations: N/A, not available

**Supplementary Information (SI) 1** Radiological findings of ten patients with fibrodysplasia ossificans progressiva (FOP). Imaging studies include plain radiographs, magnetic resonance imaging (MRI), computed tomography (CT), ultrasonography, and SPECT/CT, performed at different time points during the disease course. The table summarizes skeletal abnormalities, heterotopic ossification patterns, axial/thoracic involvement, appendicular skeletal findings, and central nervous system abnormalities identified on imaging. Radiological findings represent observations at the time of imaging and may not fully correspond to clinical findings due to the progressive and dynamic nature of FOP.
