# Supplementary material for: Fibrodysplasia ossificans progressiva in children: diagnostic pitfalls and ACVR1 genotype–phenotype spectrum
Source: Eur J Pediatr. 2026 May 2;185(5):335. doi: 10.1007/s00431-026-06974-8 (PMC13135564; doi:10.1007/s00431-026-06974-8)
Supplement: Supplementary file 2 — (DOCX 1.59 MB) [file 431_2026_6974_MOESM2_ESM.docx]

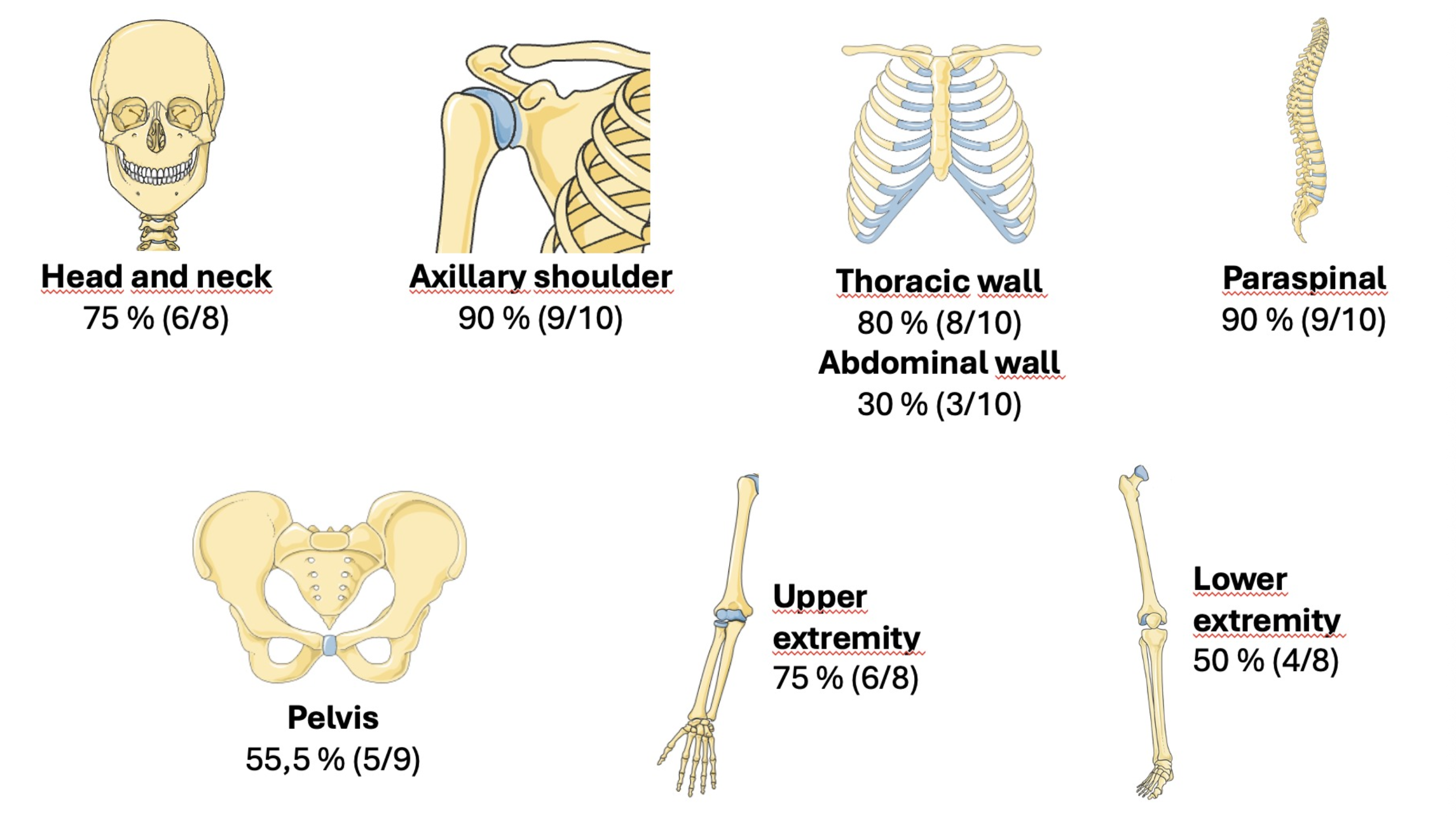


**Supplementary Information (SI) 2** Involved regions refer to anatomical areas affected by heterotopic ossification (HO) and/or inflammatory soft-tissue swellings.
