# Supplementary material for: Fibrodysplasia ossificans progressiva in children: diagnostic pitfalls and ACVR1 genotype–phenotype spectrum
Source: Eur J Pediatr. 2026 May 2;185(5):335. doi: 10.1007/s00431-026-06974-8 (PMC13135564; doi:10.1007/s00431-026-06974-8)
Supplement: Supplementary file 3 — (DOCX 23.9 KB) [file 431_2026_6974_MOESM3_ESM.docx]

| Supplementary Information (SI) 3. Key clinical, demographic, diagnostic, and neuroradiological features of previously reported Turkish patients with fibrodysplasia ossificans progressiva (FOP). | | | | | | | | |
| --- | --- | --- | --- | --- | --- | --- | --- | --- |
| **Feature** | **Doğan et al. 2023 (15)** | **Yazıcıoğlu et al. 2013 (16)** | | | | **Civan et al. 2018 (17)** | **Atik et al. 2015 (18)** | **Aksoy et al. 1998 (19)** |
| **Patient** | P1 | P2 | P3 | P4 | P5 | P6 | P7 | P8 |
| **Age at symptom onset / Initial symptoms** | 4 years / Soft-tissue swellings | 3 years / Heterotopic ossification | 4 years / Heterotopic ossification | 14 years / Heterotopic ossification | 10 years / Heterotopic ossification | 5 years / Trauma-induced soft-tissue swellings and limitation of joint mobility | 5 years / Trauma-induced soft-tissue swellings and limitation of joint mobility | N/A |
| **Age at first reported evaluation** | 20 years | 13 years | 13 years | 23 years | 25 years | 5 years | 5 years | 8 years |
| **Sex** | F | F | F | F | M | M | F | M |
| **Parental consanguinity** | N/A | N/A | N/A | N/A | N/A | N/A | - | N/A |
| **Height (cm, SDS)** | N/A | N/A | N/A | N/A | N/A | N/A | 109 cm (-0.24 SDS) | 107 cm (-3.73 SDS) |
| **Weight (kg, SDS)** | N/A | N/A | N/A | N/A | N/A | N/A | 18 kg (0.23 SDS) | 16.5 kg (-2.77 SDS) |
| **HC (cm, SDS)** | N/A | N/A | N/A | N/A | N/A | N/A | N/A | N/A |
| **Hallux valgus and/or phalangeal dysmorphism** | + | + | + | + | - | + | + | + |
| **Heterotopic ossification** | + | + | + | + | + | + | + | + |
| **Soft-tissue swellings** | + | N/A | N/A | N/A | + | + | + | + |
| **Scalp nodules** | + | N/A | N/A | N/A | N/A | N/A | N/A | N/A |
| **Osteochondromas** | + | N/A | N/A | N/A | N/A | N/A | N/A | + |
| **Dental/Oral findings** | N/A | N/A | N/A | N/A | Limited TMJ mobility | N/A | - | N/A |
| **Scoliosis** | + | N/A | N/A | N/A | N/A | - | - | + |
| **Chest deformities** | N/A | N/A | N/A | N/A | N/A | - | + | N/A |
| **Distal limb reduction defects** | - | - | - | - | - | - | - | - |
| **Restricted neck mobility** | + | N/A | N/A | N/A | N/A | + | + | + |
| **Limitation of joint mobility** | + | N/A | N/A | N/A | + | N/A | + | + |
| **Hearing loss** | N/A | N/A | N/A | N/A | N/A | N/A | N/A | N/A |
| **Renal stones** | N/A | N/A | N/A | N/A | N/A | N/A | N/A | N/A |
| **Cognitive state** | N | N/A | N/A | N/A | N/A | N/A | N/A | N/A |
| **Dysmorphic facial features** | N/A | N/A | N/A | N/A | N/A | N/A | Broad forehead, large low-set posteriorly positioned ears, trapezoid philtrum, short neck | N/A |
| **Radiological findings** | - | N/A | N/A | N/A | N/A | Fusion anomalies at the posterior elements of cervical and upper thoracic spine, bilateral short broad femoral necks | MRI: T2-hyperintense, T1-hypointense lesion in the left cervicothoracic paravertebral muscles and fascial planes with marked volume increase | CT: Ossifications in the bilateral upper extremities and posterior thoracic extrathoracic soft tissues |
| **Biopsy** | N/A | N/A | N/A | N/A | N/A | Irregular collagen and fibroblastic proliferation | - | Osteochondroma |
| **Other findings** | Epilepsy, secondary amenorrhea, uterus didelphys, right uterine horn stenosis, fundal myoma uteri, left ovarian follicle cyst | Mitral valve prolapse | Mitral valve prolapse | Left eye heterochromia | Sparse, thin scalp hair (more prominent in second decade) | - | - | Hirsutism |
| ***ACVR1 variant (NM_001111067.4)*** | N/A | c.617G>A p.Arg206His | c.617G>A p.Arg206His | c.617G>A p.Arg206His | c.774G>T p.Arg258Ser | c.617G>A p.Arg206His | c.617G>A p.Arg206His | N/A |
| **Segregation analysis** | N/A | *De novo* | *De novo* | *De novo* | *De novo* | N/A | *De novo* | N/A |

Abbreviations: CT, computed tomography; F, female; FOP, fibrodysplasia ossificans progressiva; HC, head circumference; M, male; MRI, magnetic resonance imaging; N, normal; N/A, not available; SDS, standard deviation score; TMJ, temporomandibular joint

**Supplementary Information (SI) 3** Key clinical, demographic, diagnostic, and neuroradiological features of previously reported Turkish patients with fibrodysplasia ossificans progressiva (FOP).
